# Insights Into Creutzfeldt-Jakob Disease With a Case Series From a District General Hospital and a Literature Review

**DOI:** 10.7759/cureus.92026

**Published:** 2025-09-10

**Authors:** Balwant Rai, Shalini Nandish, Marc Randall, Anu Rajgopal

**Affiliations:** 1 Radiology, Calderdale and Huddersfield NHS Foundation Trust, Huddersfield, GBR; 2 Neurology, Leeds Teaching Hospitals NHS Trust, Huddersfield, GBR; 3 Pathology, Calderdale and Huddersfield NHS Foundation Trust, Huddersfield, GBR

**Keywords:** cortex involvement, creutzfeldt–jakob disease, diffusion-weighted imaging (dwi), flair hyperintensity, magnetic resonance imaging (mri), myoclonus, periodic sharp wave complexes, prion disease, rapidly progressive dementia, rt-quic

## Abstract

Creutzfeldt-Jakob disease (CJD) is a rare, rapidly progressive, and invariably fatal neurodegenerative disorder caused by misfolded prion proteins accumulating in the central nervous system. Sporadic CJD (sCJD), the most common subtype, typically presents with rapidly progressive dementia, myoclonus, ataxia, and akinetic mutism. Radiological imaging, particularly diffusion-weighted MRI, has emerged as a cornerstone in the early diagnosis of sCJD, often identifying abnormalities before other tests become positive. This case series presents five patients diagnosed with sCJD at a single district general hospital, including a rare cluster of three cases within one calendar year. Each case highlights the crucial role of MRI in diagnosing CJD. In all cases, MRI demonstrated cortical and/or deep grey matter diffusion restriction with corresponding variable fluid-attenuated inversion recovery (FLAIR) signal changes, most prominently involving the frontoparietal and occipital lobes or basal ganglia. EEG and cerebrospinal fluid (CSF) analysis, including real-time quaking-induced conversion (RT-QuIC), supported the clinical diagnosis. This series highlights the importance of timely neuroimaging in the diagnostic pathway of sCJD and underscores the role of radiology in facilitating early specialist referral, infection control measures, and accurate disease classification. This series also highlights the importance of multidisciplinary collaboration and the need for vigilance, even in general hospital settings, as imaging can detect otherwise overlooked or atypical cases. Early radiological identification not only aids in confirming diagnosis but also plays a key role in differentiating CJD from potentially reversible mimics, ensuring appropriate patient management.

## Introduction

Creutzfeldt-Jakob disease (CJD) is a rare, rapidly progressive, and invariably fatal neurodegenerative disorder classified within the transmissible spongiform encephalopathies (TSEs). The disease results from the pathological accumulation of misfolded prion proteins (PrPSc) in the central nervous system, which arise from a conformational change in the normal cellular prion protein (PrPC). These misfolded proteins propagate by inducing similar structural changes in native prion proteins, initiating a self-amplifying cascade. Notably, PrPSc is highly resistant to proteolytic degradation and conventional sterilisation methods, enabling its persistence in neural tissue and contributing to extensive neuropathological damage. Hallmark histopathological findings include spongiform vacuolation, neuronal loss, astrocytosis, and synaptic degeneration. Clinically, CJD is characterised by rapidly progressive dementia, behavioural and psychiatric disturbances, cerebellar ataxia, myoclonus, and visual impairment, culminating in akinetic mutism and death, typically within months of onset.

Although CJD is extremely rare [1], with a global incidence estimated at one to two cases per million population annually, its impact is profound due to its rapid progression, lack of effective therapy, and fatal outcome. In the United Kingdom, surveillance data from the National CJD Research and Surveillance Unit (NCJDRSU) indicate that the annual incidence of sporadic CJD (sCJD) is approximately 1.6 cases per million [2], mirroring global epidemiological patterns. Most cases are sporadic, while a smaller proportion are attributable to pathogenic variants in the PRNP gene or acquired forms linked to iatrogenic transmission and dietary exposure to BSE. Despite decades of research, there is currently no curative or disease-modifying treatment, and management remains supportive. Given its clinical severity, rapid course, and infection control implications, early recognition and accurate diagnosis of CJD are essential both for patient care and public health safety.

## Case presentation

We present a case series of five CJD cases (Table 1) identified within a single district general hospital (DGH), including a rare clustering of three cases within a single calendar year. All cases were proven to be sporadic in nature, with no history of recent neurosurgical procedures, no shared residential address, and no familial relationship, making this clustering particularly unusual. This rare concentration of cases underscores the importance of clinical and radiological vigilance, particularly radiology's crucial role in the early detection of CJD.

**Table 1:** Case summary - Creutzfeldt-Jakob disease (CJD) CSF: cerebrospinal fluid; DWI: diffusion-weighted imaging; EEG: electroencephalogram; FLAIR: fluid-attenuated inversion recovery; MM: methionine/methionine homozygosity at codon 129 of the PRNP gene; PRNP: prion protein gene; RT-QuIC: real-time quaking-induced conversion; sCJD: sporadic Creutzfeldt-Jakob disease

Case	Age/sex	Clinical features	EEG findings	MRI findings	Final diagnosis
1	60/F	Confusion, rapidly progressive cognitive decline, myoclonus	Bilateral semi-periodic (right more than left side) sharp wave discharges	Cortical ribboning (restricted diffusion + FLAIR hyperintensity). Basal ganglia, thalamus, and cerebellum normal.	sCJD (CSF RT-QuIC positive)
2	35/F	Rapid cognitive decline, rigidity, ataxia, myoclonus	Asymmetrical slow wave activity with no typical periodic sharp wave or triphasic waves	Basal ganglia restricted diffusion + FLAIR hyperintensity with mild antero-posterior gradient; later Cortical ribboning. Thalamus and cerebellum normal.	sCJD (CSF RT-QuIC positive, codon 129 MM)
3	59/F	Confusion, visual changes, myoclonus, reduced mobility	Low voltage and slow background in the delta-theta range with minimal periodic sharp wave complexes of triphasic morphology	Cortical ribboning. Basal ganglia, thalamus, and cerebellum normal.	sCJD (CSF RT-QuIC positive)
4	59/M	Non-verbal, urinary incontinence, stiffness, global cognitive decline	-	Cortical ribboning. Basal ganglia, thalamus, cerebellum normal.	sCJD MM2 on post-mortem evaluation. (CSF RT-QuIC positive, no PRNP mutation)
5	56/M	Unsteadiness, dysphasia, blurred vision, partial seizure, fluctuating symptoms	Bilateral generalized periodic sharp waves of severe encephalopathy	Basal ganglia restricted diffusion + FLAIR hyperintensity with mild antero-posterior gradient; later cortical ribboning. Thalamus and cerebellum normal.	Probable sCJD (based on EEG, MRI, and clinical picture)

Case 1

**Figure 1:**
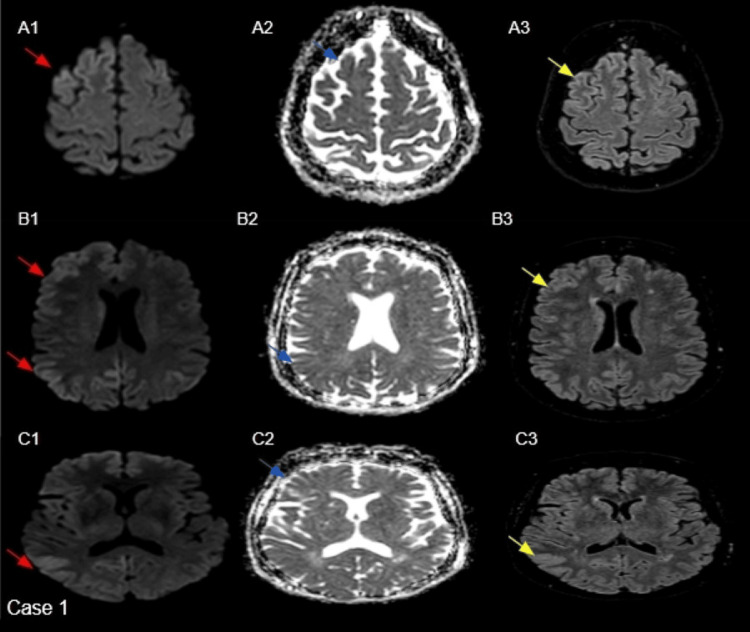
Axial B-1000 diffusion images (red arrows in A1, B1, and C1) demonstrate cortical hyperintensity involving the bilateral frontoparietal convexities, more pronounced on the right. Corresponding ADC maps (blue arrows in A2, B2, and C2) show mildly reduced signal in the same regions, and FLAIR images (yellow arrows in A3, B3, and C3) reveal subtle hyperintensity. The basal ganglia, thalami, and cerebellum appear normal. ADC: apparent diffusion coefficient; FLAIR: fluid-attenuated inversion recovery

A 60-year-old female with a three-week history of new-onset confusion and rapidly progressive cognitive impairment accompanied by myoclonic jerks. EEG demonstrated bilateral semi-periodic (right more than left) sharp wave discharges. In light of the patient's acute memory loss, progressive confusion, and characteristic EEG abnormalities, a prion disease was suspected. Subsequent MRI imaging revealed findings that were suspicious of CJD, as mentioned in Figure 1. Cerebrospinal fluid (CSF) analysis was positive for real-time quaking-induced conversion (RT-QuIC), definitively confirming the diagnosis of sCJD.

Case 2

**Figure 2:**
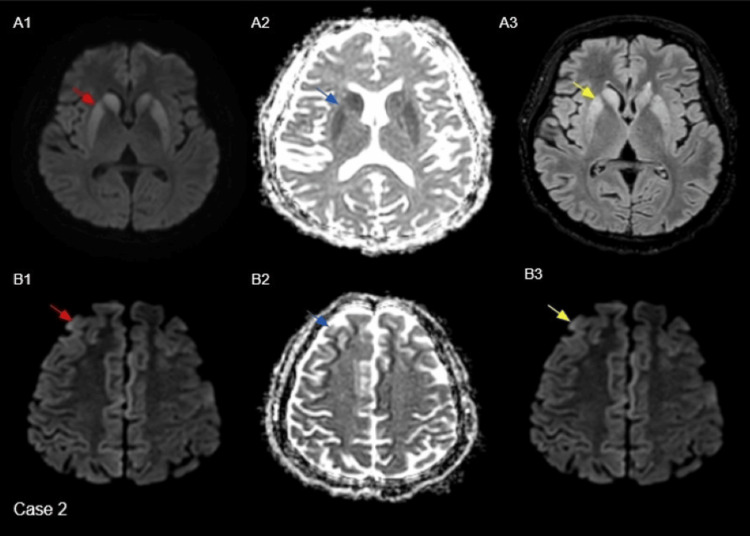
Initial MRI: Axial B-1000 diffusion images (red arrows in A1) show diffusion hyperintensity involving the bilateral striatum, demonstrating an anteroposterior gradient. Corresponding ADC maps (blue arrows in A2) reveal reduced signal in the same regions, and FLAIR images (yellow arrows in A3) show matching hyperintensity. Follow-up MRI after 20 days: Axial B-1000 diffusion images (red arrows in B1) demonstrate new cortical hyperintensity involving the bilateral frontal lobes. Corresponding ADC maps (blue arrows in B2) show reduced signal, and FLAIR images (yellow arrows in B3) reveal associated hyperintensity. The thalamus and cerebellum appear normal. ADC: apparent diffusion coefficient; FLAIR: fluid-attenuated inversion recovery

A 35-year-old female presented with rapidly progressive cognitive changes, notable rigidity, ataxia, and troublesome myoclonus. The EEG showed asymmetrical slow-wave activity with no typical periodic sharp waves or triphasic waves. Subsequent MRI imaging revealed findings that were suspicious of CJD, as mentioned in Figure 2. CSF analysis was positive for RT-QuIC, confirming the diagnosis of sCJD. No gene mutation was identified in the methionine homozygous status at codon 129. Unfortunately, the patient succumbed to the disease.

Case 3

**Figure 3:**
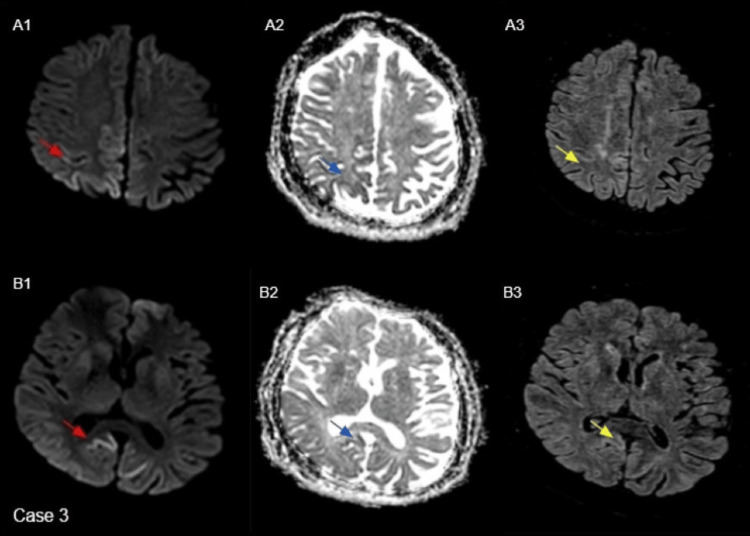
Axial B-1000 diffusion images (red arrows in A1, B1) demonstrate cortical hyperintensity involving the bilateral Parieto-occipital lobe convexities, more pronounced on the right. Corresponding ADC maps (blue arrows in A2, B2) show reduced signal in the same regions, and FLAIR images (yellow arrows in A3, B3) reveal subtle hyperintensity. The basal ganglia, thalami, and cerebellum are normal. ADC: apparent diffusion coefficient; FLAIR: fluid-attenuated inversion recovery

A 59-year-old female presented with a worsening reduction in mobility, confusion, and visual changes. Over time, she experienced increasing jerks and twitches in her arms and legs. The EEG showed low voltage and a slow background in the delta-theta range, with minimal periodic sharp wave complexes of triphasic morphology. Some of these were sharpened in nature and triphasic in morphology. Findings suggested bi-hemispheric brain dysfunction. Subsequent MRI imaging revealed findings suspicious of CJD, as shown in Figure 3. CSF analysis was positive for RT-QuIC, confirming the diagnosis of sCJD. Unfortunately, the patient succumbed to the disease.

Case 4

**Figure 4:**
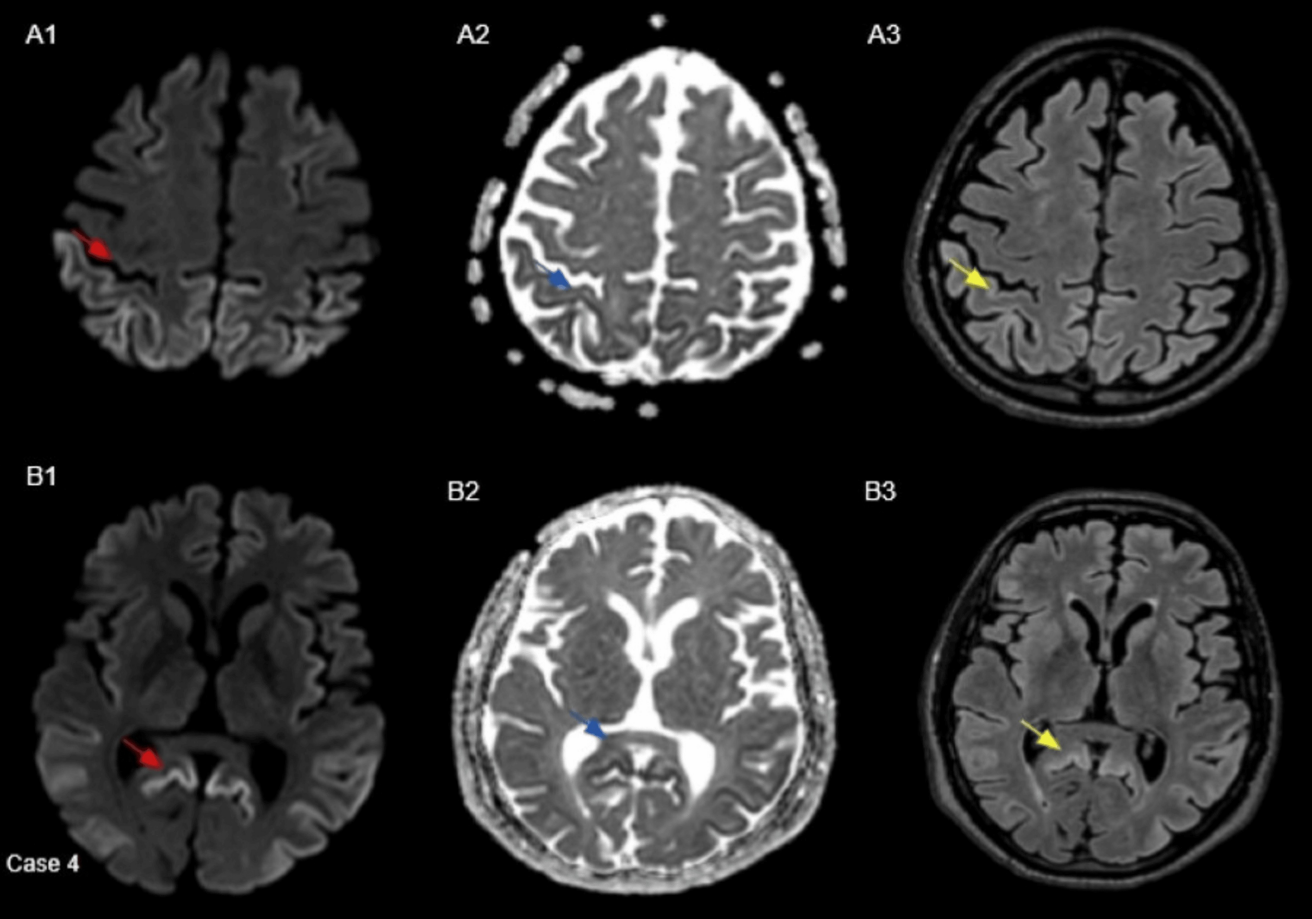
Axial B-1000 diffusion images (red arrows in A1, B1) demonstrate cortical hyperintensity involving the bilateral parieto-occipital lobe convexities, more pronounced on the right. Corresponding ADC maps (blue arrows in A2, B2) show reduced signal in the same regions, and FLAIR images (yellow arrows in A3, B3) reveal cortical hyperintensity. The basal ganglia, thalami, and cerebellum were unremarkable. ADC: apparent diffusion coefficient; FLAIR: fluid-attenuated inversion recovery

A 59-year-old male was non-verbal for nine weeks, incontinent of urine, and experiencing muscle stiffness, with progressive general decline. Subsequent MRI imaging revealed findings suspicious of CJD, as mentioned in Figure 4. CSF analysis was positive for RT-QuIC, confirming the diagnosis of sCJD. Unfortunately, the patient succumbed to the disease. Biochemical analysis and codon 129 assessment on a post-mortem sample revealed the subtype to be MM type 2, and PRNP sequencing did not detect any mutations in the PRNP gene. Therefore, the final diagnosis was sCJD MM2. 

Case 5

**Figure 5:**
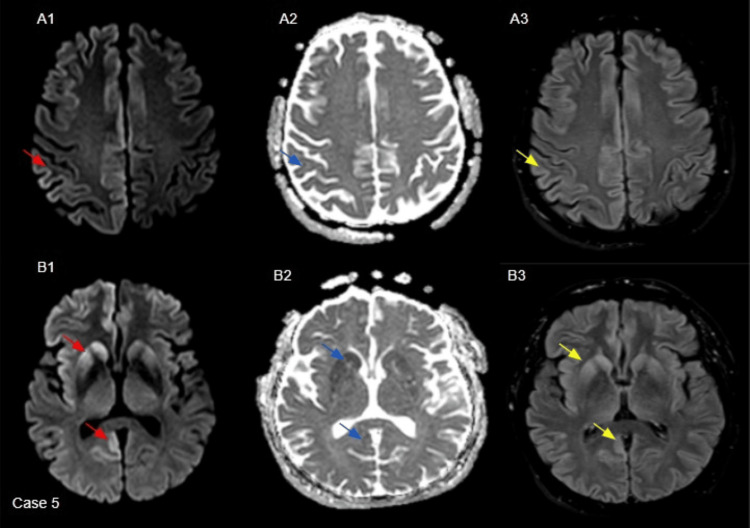
Axial B-1000 diffusion images (red arrows in A1, B1) demonstrate cortical hyperintensity involving the bilateral frontal, parieto-occipital lobe convexities, more pronounced on the right and striatum with anteroposterior gradient. Corresponding ADC maps (blue arrows in A2, B2) show reduced signal in the same regions, and FLAIR images (yellow arrows in A3, B3) reveal cortical hyperintensity. The thalami and cerebellum were unremarkable. ADC: apparent diffusion coefficient; FLAIR: fluid-attenuated inversion recovery

A 56-year-old male presented with unsteadiness, dysphasia, and blurred vision. The patient exhibited fluctuating neurological symptoms while hospitalised and experienced a partial seizure. EEG demonstrated bilateral and generalised periodic sharp wave discharges from both hemispheres. The clinical picture and EEG findings were consistent with a probable sCJD diagnosis. Subsequent MRI imaging findings were suspicious of CJD, as mentioned in Figure 5. Unfortunately, the patient succumbed to the disease.

## Discussion

CJD arises from the conformational changes of the normal cellular prion protein (PrPC) into a misfolded, pathogenic isoform (PrPSc). This abnormal protein accumulates in neural tissue, causing characteristic spongiform degeneration, neuronal loss, and reactive astrogliosis. In specific subtypes - particularly variant CJD (vCJD) and Gerstmann-Sträussler-Scheinker syndrome - amyloid plaque deposition is also present [3].

Depending on the cause and clinical characteristics, CJD is divided into four main types: iatrogenic (iCJD), genetic (gCJD), sCJD, and vCJD [4].

sCJD is subclassified based on the polymorphism at codon 129 of the prion protein gene (methionine (M) or valine (V)) and the size of protease-resistant PrPSc (type 1 at 21 kDa or type 2 at 19 kDa). These molecular subtypes correlate with clinical variants [5], such as MM1/MM2 cortical (Heidenhain variant, characterised by visual symptoms) and VV2 (Brownell-Oppenheimer variant, characterised by early cerebellar involvement).

Different forms of CJD exhibit distinct features. The most common, sCJD, typically progresses rapidly, with a median survival of about six months. vCJD is characterised by early psychiatric symptoms, slower progression of approximately 12-14 months, and predominantly affects younger patients, with a median age of around 28 years. vCJD was of particular concern in the late 1990s and early 2000s due to exposure to bovine spongiform encephalopathy (BSE) in the United Kingdom; however, it is now extremely rare, with only a few isolated cases reported in recent years [6]. gCJD requires confirmation through genetic testing, may manifest at an earlier age, often resembles sCJD clinically, and accounts for roughly 10% of cases [7]. It is caused by inherited mutations in the PRNP gene, and a family history is frequently present. iCJD is uncommon and is associated with exposure to contaminated medical instruments or procedures.

Clinical features

In its early phase, patients may present with nonspecific symptoms such as fatigue, behavioural or mood disturbances (e.g., depression, anxiety, insomnia), mild visual changes, or personality changes, often leading to misdiagnosis.

The hallmark feature is rapidly progressive dementia, typically developing within weeks. It is often accompanied by cerebellar signs (ataxia, nystagmus), extrapyramidal features (rigidity, bradykinesia), myoclonus, and visual disturbances. Startle-induced myoclonus and gait abnormalities are particularly characteristic of this condition. Seizures and cortical blindness are less common.

As the disease progresses, pyramidal signs and worsening motor dysfunction lead to akinetic mutism, marked by profound loss of voluntary movement and speech. Most patients die within a year, often due to respiratory complications.

Distinct clinical phenotypes exist, reflecting different patterns of brain involvement [5]. The Heidenhain variant presents with early visual symptoms due to occipital cortex involvement. The Brownell-Oppenheimer variant is characterised by predominant or isolated cerebellar ataxia. The Stern-Garcin variant manifests as presenile dementia, sleep disturbances, and extrapyramidal signs, and is associated with thalamic degeneration. The amyotrophic variant mimics motor neuron disease, with early weakness and muscle wasting.

Although these clinical phenotypes have similar histopathologic hallmarks (spongiform degeneration, neuronal loss, and gliosis) distributed in expected topography, the "expected abnormalities" are not always depicted on MR images.

Diagnostic workup and imaging

The diagnostic criteria established by the NCJDRSU [2] are most commonly used in the United Kingdom for the diagnosis of CJD, as detailed in Table 2.

**Table 2:** Diagnostic criteria for CJD (NCJDRSU, Edinburgh) CJD: Creutzfeldt-Jakob disease; CSF: cerebrospinal fluid; DWI: diffusion-weighted imaging; EEG: electroencephalography; FLAIR: fluid-attenuated inversion recovery; MRI: magnetic resonance imaging; NCJDRSU: National Creutzfeldt-Jakob Disease Research and Surveillance Unit, Edinburgh; RT-QuIC: real-time quaking-induced conversion

Diagnostic criteria	
Definite CJD	Progressive neuropsychiatric syndrome and confirmation by neuropathology, immunocytochemistry, or biochemical tests.
Probable CJD	Criterion I + at least two features from Criterion II + one or more of the following: typical EEG (periodic sharp wave complexes); typical MRI (restricted diffusion in caudate, putamen, or ≥2 cortical regions on DWI/FLAIR); positive CSF 14-3-3 protein; positive CSF/tissue RT-QuIC and exclusion of other causes after thorough workup.
Possible CJD	Criterion I + at least two features from Criterion II + duration < 2 years + inconclusive EEG/MRI/CSF/RT-QuIC.
Criterion I	Core symptom: rapidly progressive cognitive impairment.
Criterion II	Supporting features (≥2 required): myoclonus; visual or cerebellar disturbances; pyramidal or extrapyramidal signs; akinetic mutism

Clinical criteria are defined as rapidly progressive dementia plus at least two of the following four clinical features: myoclonus, visual disturbance or cerebellar dysfunction, pyramidal or extrapyramidal features, and akinetic mutism. Alternative diagnoses and mimicking disorders must be excluded. 

In CJD, periodic sharp-wave complexes (PSWCs) at a frequency of approximately 1 Hz are considered a distinctive EEG pattern that typically appears in the middle to late stages of the illness. These results are a helpful but not conclusive diagnostic sign, with a reported sensitivity of about 64% and a high specificity of 91% [8]. 

Several CSF biomarkers are essential in the diagnosis of sCJD. The 14-3-3 protein is the most often investigated and has been identified as a distinctive and suggestive biomarker for sCJD. The presence of 14-3-3 protein in CSF, however, appears to indicate only significant brain tissue destruction, as seen in sCJD, and it is also present in ischaemic stroke and meningoencephalitis [9]. 

CSF RT-QuIC is a highly disease-specific biomarker for CJD, with studies demonstrating a diagnostic specificity of 99% to 100%. The sensitivity of the test depends on both the assay's manufacturing process and the molecular subtype of the disease. First-generation RT-QuIC assays have sensitivity ranges of 73% to 89%, but second-generation assays have heightened sensitivity ranges of 92% to 97%. The most common molecular subtypes of sCJD, including MM1, MV1, and VV2, have excellent diagnostic performance despite a somewhat lower sensitivity reported in MV2 cases (75-93%) [10]. 

CJD can be caused by mutations in the prion protein gene (PRNP) on chromosome 20p12-pter. These pathogenic variants account for around 10-15% of all cases of CJD and are typically associated with a family history that indicates autosomal dominant inheritance with variable penetrance. However, the use of genetic testing in prion monitoring systems worldwide has increased. As a result, it has been surprising to find PRNP mutations in individuals who present with a clinical picture comparable to sCJD but have no clear family history. Significant phenotypic variability is seen in hereditary prion diseases, which often share clinical and pathological features with sCJD [11]. 

MRI

Detectable MRI imaging findings, particularly those obtained from diffusion-weighted imaging (DWI), can arise in sCJD patients before the development of clinical symptoms, even in undiagnosed cases with ordinary or unusual results on EEG and CSF evaluation [5]. As a result, the method is essential for promoting early detection. 

Magnetic resonance imaging (MRI) plays a critical role in diagnosing CJD. It reveals both classical and nonclassical patterns that offer crucial diagnostic and pathophysiological insights [5]. 

Classical MRI findings involve the cerebral cortex, basal ganglia, and occasionally the cerebellum. The cerebral cortex may show focal or diffuse hyperintensities, often asymmetric, with sparing of the perirolandic region. These signal changes can fluctuate, and DWI is the most sensitive sequence, supported by apparent diffusion coefficient (ADC) and fluid-attenuated inversion recovery (FLAIR). In the basal ganglia, particularly the caudate and putamen, symmetric or asymmetric involvement with an anterior-to-posterior gradient is commonly seen. Signal intensity increases with disease progression. Cerebellar involvement is typically seen as atrophy in later stages, with DWI hyperintensities being relatively uncommon.

In addition to these classical findings, several nonclassical MRI features have been described in CJD. Hyperintensities in the posterior thalami (pulvinar sign) and dorsomedial thalami (double hockey stick sign) - more typical of vCJD-can appear in up to 45% of sCJD cases. T1 hyperintensity in the globus pallidus has also been noted, likely from prion accumulation, though paradoxically often absent in the putamen, possibly due to spongiform degeneration masking the signal. 

Imaging patterns in sCJD may correlate with distinct clinical phenotypes, although they are not always observed. The Heidenhain variant is characterised by isolated visual symptoms at onset, including poor vision, hallucinations, and distorted colour perception. EEG typically demonstrates PSWCs, and CSF biomarkers such as 14-3-3 and t-tau are elevated. MRI often shows occipitoparietal hyperintensity on DWI or FLAIR in approximately 80% of cases, with most patients exhibiting the MM1 molecular subtype [12]. In contrast, the Brownell-Oppenheimer variant presents primarily with isolated cerebellar ataxia. EEG frequently lacks positive sharp waves and slow waves, and CSF 14-3-3 protein may not be elevated. Imaging findings commonly include cerebellar atrophy, increased ADC, and involvement of the basal ganglia and cortex on DWI, and this form is most often associated with the VV2 molecular subtype [13]. The Stern variant is distinguished by prominent behavioural and sleep disturbances, such as insomnia, frequent arousals, and enacted dreams, often in younger individuals. EEG does not show PSWCs, and CSF findings are usually normal. Imaging typically reveals hypoperfusion or hypometabolism on SPECT or PET, with no signal alterations on DWI, and histopathologic analysis demonstrates extensive thalamic degeneration [14].

In 2020, a study investigating a large cohort of patients with definite sCJD found that DWI MRI was associated with an improved diagnostic index, yielding 92% sensitivity and 97% specificity [15].

Imaging mimics include postictal abnormalities, metabolic encephalopathy, acute hypoxic/ischemic encephalopathy, and autoimmune or infectious encephalitis. It is necessary to carefully differentiate these differential diagnoses from CJD by utilising imaging characteristics, laboratory testing (such as metabolic panels, serology, and CSF analysis), and clinical context. 

The earliest detectable abnormalities in our case series were frequently DWI hyperintensities, accompanied by corresponding FLAIR signal changes and ADC restriction. Radiological diagnosis often enabled the clinical team to consult a neurologist for a detailed evaluation, microbiological consultation, and referral to NCJDRSU for a prompt diagnosis. The high sensitivity of DWI in identifying early cortical and deep grey matter involvement in sCJD, as well as its usefulness in distinguishing phenotypes according to anatomical predilections and progression patterns, is further demonstrated by this case study. 

A review of the literature [16-17], including five case series from the UK and worldwide, demonstrates that sCJD typically affects older adults, with median ages ranging from 66 to 77 years across various studies. Common clinical features reported include rapidly progressive dementia, psychiatric or behavioural disturbances, myoclonus, and gait ataxia. MRI findings are consistently dominated by cortical ribboning and basal ganglia hyperintensities, while EEG often shows PSWC, sometimes accompanied by generalised diffuse slowing. Details of these literature findings are summarised in Table 3. In comparison, our case series of five patients had a slightly younger median age of 59 years, with clinical presentation predominantly featuring rapid cognitive decline and myoclonus. Radiologically, our patients showed similar patterns of cortical ribboning and basal ganglia involvement, reflecting the classical imaging findings reported in the literature. EEG findings in our cohort, however, were more variable, showing generalised or diffuse slowing with only intermittent or absent PSWC, as shown in larger published series. Overall, our series aligns with the established radiological and clinical hallmarks of sCJD, while also emphasising that younger patients may present with less typical EEG features.

**Table 3:** Salient points from the literature review of recent sporadic Creutzfeldt-Jakob disease (sCJD) and comparison with our case series

Study	Total patients	Median age (years)	Most common clinical features	Most common MRI features	Most common EEG features
Tam et al. [16]	1178	68	Psychiatric/behavioural disturbance, dementia, and myoclonus	Cortical ribboning and basal ganglia changes.	Periodic sharp wave complexes, but not specific for sCJD.
Jesuthasan et al. [17]	102	66	Not specified	Cortical ribboning and basal ganglia changes.	Periodic sharp wave complexes (PSWC) and generalised diffuse slow waves
Dumrikarnlert et al. [18]	18	66	Dementia, myoclonus	Cortical ribboning and basal ganglia changes.	PSWC and generalised diffuse slow waves
Rus et al. [19]	9	72	Rapid dementia, gait ataxia	Cortical ribboning and basal ganglia changes.	PSWC
Rong et al. [20]	5	77	Rapid cognitive decline	Cortical ribboning and basal ganglia changes.	PSWC
Our case series	5	59	Rapid cognitive decline and myoclonus	Cortical ribboning and basal ganglia changes.	Generalised/diffuse slowing with variable presence of periodic sharp wave complexes

There is currently no cure for CJD, which remains uniformly lethal, with a median survival of about seven months from symptom onset. The cornerstone of care remains supportive, requiring close collaboration between radiologists and clinical teams. From a public health perspective, CJD is a notifiable disease in many countries, including the USA, UK, Australia, and the European Union (EU). 

## Conclusions

Early recognition of CJD is crucial, not only for timely patient counselling and care planning but also for infection control and public health notification. Radiologists, particularly through the use of diffusion-weighted MRI, showing cortical ribboning and variable basal ganglia involvement, play a central role in raising early diagnostic suspicion. In all cases in our series, radiology played a significant role, guiding the decision for CSF evaluation with adequate preparation and precautions, and leading to the diagnosis and timely notification of the disease. This case series highlights the diagnostic value of neuroimaging in a DGH, where specialist neuroradiology input may be limited and patients often first present to general medical services.

Given the diagnostic complexity and the potential overlap with treatable mimics, close multidisciplinary collaboration is essential. Prompt communication with the NCJDRSU and the National Prion Clinic (NPC) is recommended when CJD is suspected. Early coordination with the local microbiology laboratory is also necessary to expedite CSF sample processing for RT-QuIC testing, which requires strict handling and transport protocols. Overall, this series emphasises the importance of radiologist vigilance at the DGH level, the value of early imaging in atypical neurocognitive presentations, and the need for integrated, team-based pathways for rare but critical diagnoses such as CJD.
